# Ultra-low-cost mechanical smartphone attachment for no-calibration blood pressure measurement

**DOI:** 10.1038/s41598-023-34431-1

**Published:** 2023-05-29

**Authors:** Yinan Xuan, Colin Barry, Jessica De Souza, Jessica H. Wen, Nick Antipa, Alison A. Moore, Edward J. Wang

**Affiliations:** 1 Electrical and Computer Engineering Department, UC San Diego, 9500 Gilman Dr., La Jolla, CA 92093 USA; 2 Department of Medicine, UC San Diego, 9500 Gilman Dr., La Jolla, CA 92093 USA; 3 The Design Lab, UC San Diego, 9500 Gilman Dr., La Jolla, CA 92093 USA

**Keywords:** Engineering, Biomedical engineering

## Abstract

We propose an ultra-low-cost at-home blood pressure monitor that leverages a plastic clip with a spring-loaded mechanism to enable a smartphone with a flash LED and camera to measure blood pressure. Our system, called BPClip, is based on the scientific premise of measuring oscillometry at the fingertip to measure blood pressure. To enable a smartphone to measure the pressure applied to the digital artery, a moveable pinhole projection moves closer to the camera as the user presses down on the clip with increased force. As a user presses on the device with increased force, the spring-loaded mechanism compresses. The size of the pinhole thus encodes the pressure applied to the finger. In conjunction, the brightness fluctuation of the pinhole projection correlates to the arterial pulse amplitude. By capturing the size and brightness of the pinhole projection with the built-in camera, the smartphone can measure a user’s blood pressure with only a low-cost, plastic clip and an app. Unlike prior approaches, this system does not require a blood pressure cuff measurement for a user-specific calibration compared to pulse transit time and pulse wave analysis based blood pressure monitoring solutions. Our solution also does not require specialized smartphone models with custom sensors. Our early feasibility finding demonstrates that in a validation study with N = 29 participants with systolic blood pressures ranging from 88 to 157 mmHg, the BPClip system can achieve a mean absolute error of 8.72 and 5.49 for systolic and diastolic blood pressure, respectively. In an estimated cost projection study, we demonstrate that in small-batch manufacturing of 1000 units, the material cost is an estimated $0.80, suggesting that at full-scale production, our proposed BPClip concept can be produced at very low cost compared to existing cuff-based monitors for at-home blood pressure management.

## Introduction

Hypertension, defined as systolic blood pressure (SBP) of $\ge$ 130 mmHg and/or diastolic blood pressure (DBP) $\ge$ 80 mmHg^1^, affects more than 45% of adults in the US with its prevalence increasing with age, and is the most common reason for medical office visits and the use of chronic prescription medications^2–4^. Hypertension remains the leading preventable cause of premature death and disability worldwide, killing almost eight million people every year, and is projected to increase by 60% to affect 1.6 billion adults worldwide by 2025^5^. Hypertension, beyond elevating risks of cardiovascular diseases including stroke, heart disease, chronic kidney disease, and end-stage kidney disease, has also been demonstrated to accelerate cognitive decline for middle-aged and older adults, with growing evidence that hypertension is associated with a higher risk of all-cause Mild Cognitive Impairment (MCI) and Non-Amnestic MCI. It is thought that hypertension may cause cognitive impairment through cerebrovascular diseases, such as atherosclerosis of cerebral blood vessels, and damage to the blood–brain barrier.

The key areas where low-cost BP monitors can make a significant impact are low-income and/or disadvantaged communities^6,7^. These communities often face barriers to accessing healthcare services, which can lead to a lack of preventative care and increased risk for chronic diseases like hypertension^8,9^. For example, older adults living in rural communities may face challenges in accessing hypertension screening due to limited transportation options and distance from healthcare facilities. Pregnant women, particularly those from low-income backgrounds, may also struggle to access screening for preeclampsia, a potentially life-threatening hypertensive complication of pregnancy^10^. Refugees who may have limited access to healthcare services in their host country may also benefit from a low-cost BP monitor for at-home monitoring^5,11^. Making BP monitoring more accessible and affordable to enable measurements outside of the traditional clinics can also facilitate community-based hypertension management approaches proven to be effective for vulnerable populations^12–14^.

In an effort to increase accessibility to this vital measurement, we introduce *BPClip*, an ultra-low cost universal smartphone attachment that enables smartphones to measure blood pressure. The insight that enables our innovation is the use of computational imaging that combines a smartphone camera with a cheap plastic clip that employs a spring-loaded mechanism. This clip device, which has no electronic components, is compatible with any smartphone featuring a low-resolution (2 MegaPixels) camera and a light source, attributes found in virtually every modern smartphone, even the least expensive models. The basic functioning principle of the clip is called oscillometry. Oscillometry is a measurement of the arterial pulse amplitude as a function of applied pressure. Oscillometry is the basis of automated electronic BP cuffs commonly in use today and the preferred technique of the American Heart Association (AHA) and American Medical Association (AMA)^1,15^. Our system, called BPClip, performs oscillometry at the fingertip to measure BP. To enable a smartphone to measure the pressure applied to the digital artery, a moveable pinhole projection moves closer to the camera as the user presses down on the clip with increased force. As a user presses on the device with increased force, the spring-loaded mechanism compresses. In this way, the size of the pinhole encodes the pressure applied to the finger. In conjunction, the brightness fluctuation of the pinhole projection correlates to the arterial pulse amplitude. By capturing the size and brightness of the pinhole projection with the built-in camera, the smartphone can simultaneously measure the pressure applied to the finger and the resulting pulse amplitude to calculate a user’s BP with nothing but a low-cost, plastic clip and a smartphone app.

Numerous cuffless-optical solutions have been proposed for app-based and wearable BP monitoring, but all have failed to reach commercial viability due to the need for onerous and frequent per-user calibration. Per-user calibration means that to use the device, a user would need to first measure their BP with a cuff-based device at least once, but typically multiple times at different BP levels. This per-user calibration is necessary for any solution that relies on pulse transit time (PTT)^16–22^ and/or pulse wave analysis (PWA)^23–25^ or deep learning methods that use PPG and demographics^26,27^ to estimate blood pressure. This is because these indirect measurements are highly dependent on individual variations of arterial stiffness, vascular structure, and arm length. In order to use these other cuffless solutions, users must still have access to blood pressure cuffs, either personally or through a clinic. Additionally, due to changes in arterial stiffness over time, per-user calibration based systems must be recalibrated over time. Due to this reliance on calibration, PTT/PWA-based calibrated cuffless blood pressure monitoring solution is only suitable in use-cases focused on convenience of frequent/continuous monitoring of blood pressure for those who already have access to existing blood pressure monitoring infrastructure. In comparison, per-user calibration is not required for oscillometric-based methods because the measurement is performed locally at the artery using applied mechanical pressure.

In an estimated cost projection study, we demonstrate that in small-batch manufacturing of 1000 units, the material cost is an estimated $0.80, suggesting that at full-scale production, our proposed BPClip concept can be produced at very low costs compared to existing cuff-based monitors for at-home blood pressure management. Prior approaches that investigated the use of smartphones for oscillometric measurement have required specific smartphone models with a pressure-sensitive screen^28^ or sensorized devices designed as a smartphone case^29^ requiring computation, communication, or sensor components. In comparison, our low-cost mechanical solution is compatible with any smartphone featuring a flash light emitting diode (LED) and a camera.

The goal of this study is to provide feasibility findings of the potential measurement capability of a low-cost blood pressure monitoring concept that does not require per-user calibration. The ultimate translational goal is to provide widespread, at-home access to blood pressure monitoring without reliance on existing clinical infrastructure. We envision that through the incorporation of ultra-low-cost monitoring capability of vitals such as blood pressure, public health programs and telehealth services can augment these low-cost services with not just virtual consultation, but an additional quantitative measurement that is enabled through a device that is at a cost suitable for large-scale distribution.

## Results

### Concept

**Figure 1:**
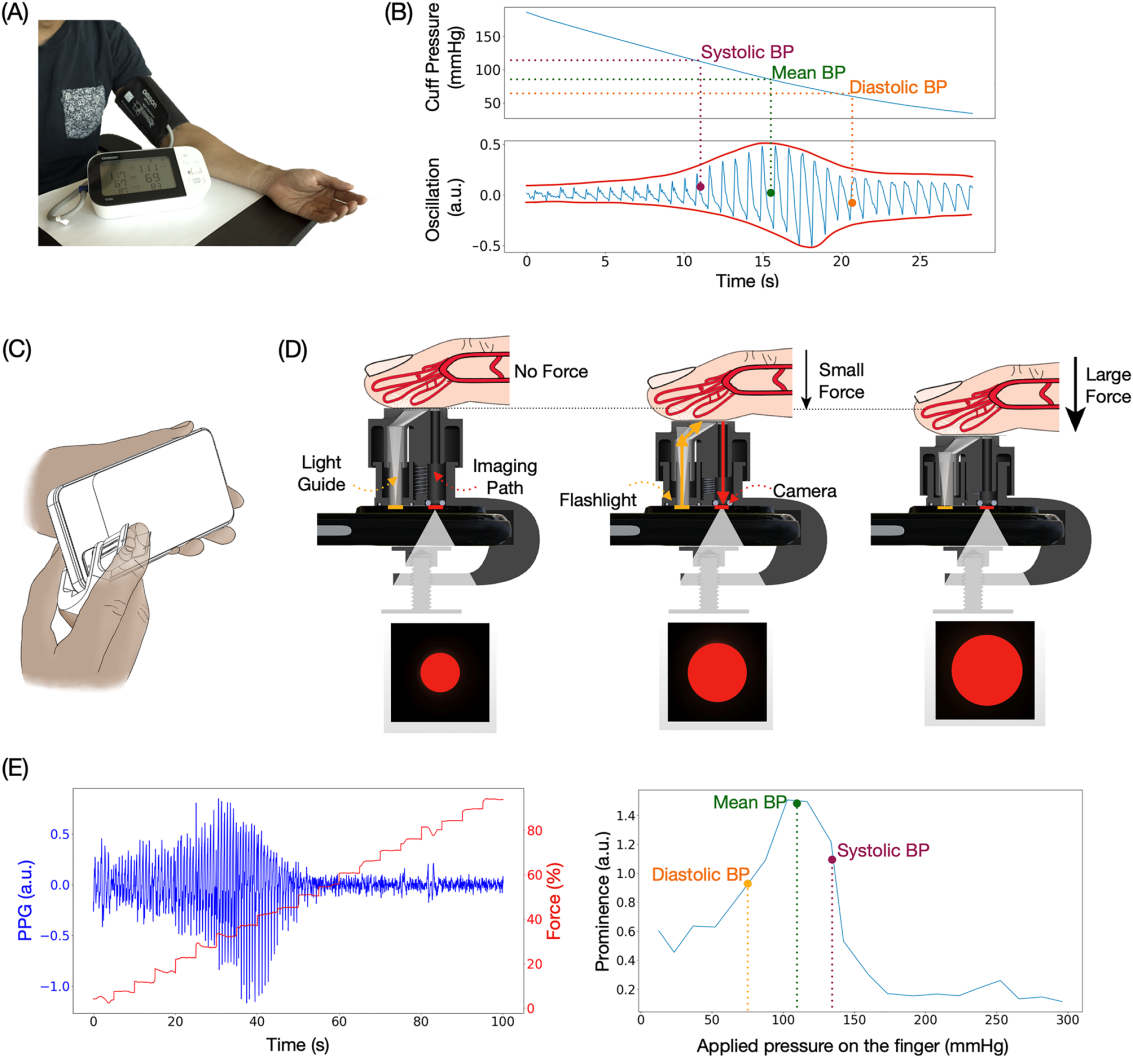
System overview: using finger oscillometry to calculate BP. (**A**) Cuff-based BP monitor. (**B**) Example of an oscillogram obtained from an automated cuff. SBP, MBP, and DBP can be calculated from the shape of the envelope of the oscillogram. (**C**) Using BPClip to measure BP. (**D**) Light emitted from the smartphone flash travels through the light guide and illuminates the finger. The reflected light travels through the imaging path via a pinhole to reach the camera, forming an image of a red circle. The pulse information is encoded by the brightness of the circle. The pressing force information is encoded by the size of the circle. As the applied force increases, the circle diameter increases. (**E**) Left: pulse and force data extracted from the red circle. Right: oscillogram reconstructed from the data on the left.

Similar to traditional automated cuff-based BP monitors, our system utilizes oscillometry to measure BP^30,31^. As such, our system does not require per-user calibration. The oscillometric method calculates BP based on the change of blood volume oscillations per heartbeat as the external pressure changes around the artery. Therefore, to use the oscillometric method, a device needs to have three features: (1) the ability to apply pressure on the artery; (2) the ability to sense how much pressure is applied to the artery; (3) the ability to measure blood volume changes as the applied pressure changes. Typically, automated blood pressure monitors exert increasing external pressure on the artery while sensing the pressure and blood volume oscillation with a pressure sensor (Fig. 1A). Figure 1B is an example of the data collected with a typical automated cuff device. The shape of the envelope of the pulse graph makes an oscillogram and is used to calculate BP^31^.

In our smartphone clip-based system, BPClip, we leverage the hardware and computational power that can be found on any modern smartphone to measure BP with the oscillometric principle by mounting a 3D-printed attachment to the smartphone’s camera. To use BPClip, the user holds the phone horizontally with the left hand and pushes down on a spring-loaded clip with the right index finger (Fig. 1C). The user then adjusts the applied force of their finger to a series of varying magnitudes as prompted by a custom smartphone user interface (Fig. 1D). This applies pressure onto the digital artery, specifically the transverse palmar arch artery. Meanwhile, the smartphone flashlight LED illuminates the fingertip by delivering light through a chrome-painted light guide (Fig. 1D). The reflected light travels back through the imaging path via a pinhole, projecting a circular image onto the camera that encodes (1) the pressure applied to the finger and (2) the volume of blood in the finger (Fig. 1D). The pressure applied to the finger is computed from the displacement of the spring-loaded attachment as it is compressed, which is reflected by the size of the circular pinhole projection on the smartphone camera image (Fig. 1D). At higher applied pressure, the pinhole is closer to the camera, creating a larger projection. The blood volume oscillation is detected by the brightness of the pinhole projection. When the applied pressure is less than the SBP, the brightness of the pinhole projection fluctuates with respect to the amount of blood through the digital artery, an effect called photoplethysmography (PPG). With more blood, more light is absorbed, resulting in low brightness and a darker projection, and with less blood, less light is absorbed, resulting in higher brightness and a brighter projection. As the applied pressure exceeds the SBP, the blood ceases to flow through the digital artery as the artery is occluded and stops the fluctuation of the pinhole projection (Fig. 1E). The smartphone application provides visual feedback guiding the user to hold their finger at increasing increments of pre-defined discrete applied forces (Fig. 3C), and extracts the applied pressure and pulse volume by tracking the size and brightness of the projection (Supplement Video 1).

### Prototype

**Figure 2:**
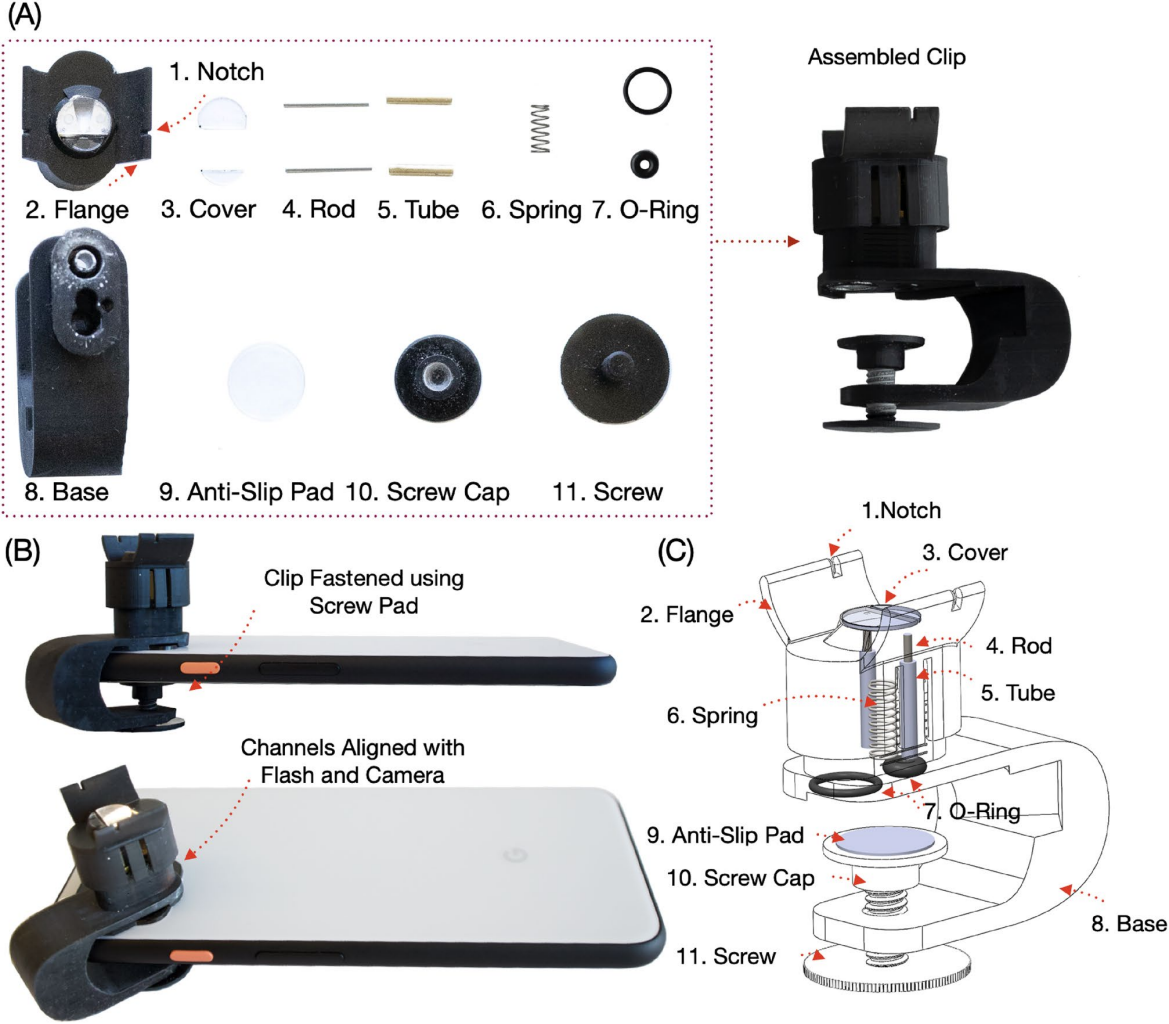
Prototype component diagram: Details of the hardware prototype. (**A**) Fully assembled BPClip and disassembled components. (**B**) BPClip mounted on a phone. (**C**) X-ray view of assembled BPClip. Legend: 1. notch to align finger; 2. flange to constrain finger angle; 3. covers to ensure a flat pressing surface; 4. rod to ensure smooth pressing; 5. tube to ensure smooth pressing; 6. spring; 7. o-rings to avoid light leakage; 8. clip base; 9. anti-slip pad; 10 and 11. clamp screw.

BPClip consists of a smartphone attachment and an Android application. The 3D-printed attachment consists of a base clip that attaches to the phone, a movable pressing platform that the finger presses, and a compression spring sandwiched in the middle (Fig. 2).

The pressing platform has two flanges that position the finger at the desired angle. On the flange, there is a notch that helps the user align their index finger with respect to the BPClip. On the pressing platform, a 1 cm diameter protrusion is centered over the linear compression spring, which acts as a uniform platform for the finger to press on and apply even pressure.

**Figure 3:**
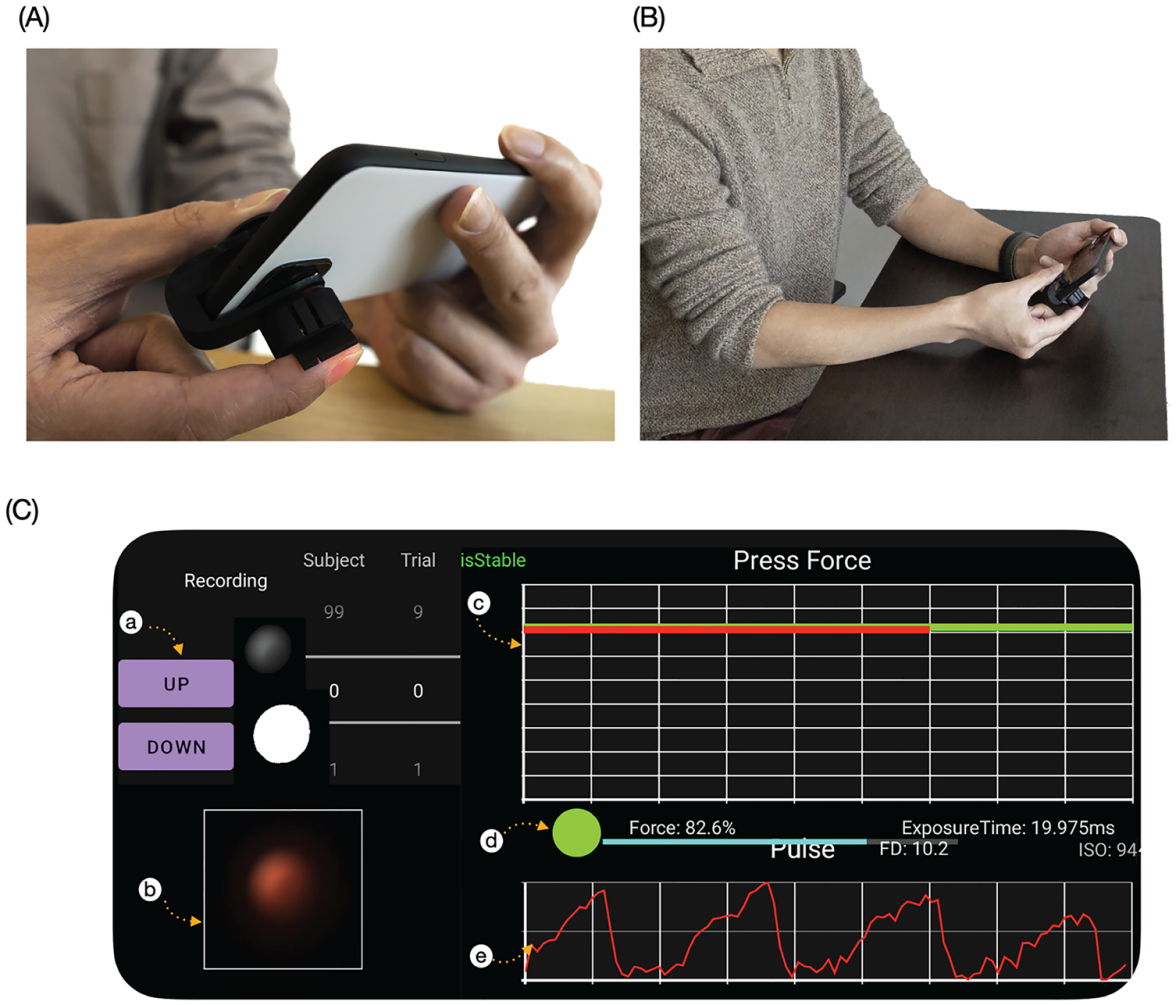
System usability: using BPClip. (**A**) The base of the right index fingernail is aligned with the notch. (**B**) The user holds the phone and clip, with BPClip at heart level. (**C**) The (user interface) UI layout of the app. Legend: a. button to record force range adjustment; b. real-time camera image preview; c. force indicator, the red line indicates the current force, while the green line indicates the target force level; d. force range indicator: the red circle indicates that the force is out of range, the yellow circle indicates that the force is within the range, and the user needs to hold for 2 more seconds to start data recording, and the green circle indicates that the data is currently being recorded with a 5-s progress bar appearing; e. real-time pulse signal display.

A light guide and an imaging path have been designed to guide the light and minimize signal attenuation and noise from light leakage (Fig. 1D). The light guide incorporates a bend to direct the light into the protrusion. This bend accommodates different designs of phones with various camera-to-flashlight distances. In our prototype, we constructed the system to support a 1 cm distance between the camera and the flash LED. To maximize light transfer from the flashlight to the finger, the inner surface of the light guide is coated with chrome paint. The imaging path is right next to the spring and directly under the 1 cm protrusion. Finally, the use of the BPClip is supported by a custom smartphone application that extracts the PPG signal and applied finger force from the camera in real time. The user interface provides users with visual feedback on the magnitude of force to apply (Fig. 3C).

The base clip is clamped onto the phone with a 3D-printed screw. A silicone pad is placed between the screw and the phone screen to ensure a non-slip interface. To ensure smooth and vertical displacement, two pairs of lubricated rods and tubes are used.

### Usage

To use BPClip, the user holds the phone in landscape mode with the left hand, with their right index finger resting on the protrusion and their right thumb resting on the base of the clip. To ensure that the camera can detect PPG signals from the transverse palmar arch artery via the pinhole, the user aligns the base of their fingernail with a notch 2 mm away from the center of the clip, and aligns the length of the finger parallel to the long axis of the clip (Fig. 3A). To eliminate hydrostatic effects, the user holds the phone at heart level, with uncrossed legs, and maintains an upright posture throughout the measurement, similar to standard protocols utilizing a cuff-based BP measurement (Fig. 3B). The user increases the pressure applied to the BPClip incrementally as guided by the on-screen visual reference (Fig. 3C). At each increment, the user holds the force for 7 s, before moving to the next force increment. Data is only recorded during the last 5 s of the total 7 s to ensure data quality. The application automatically restarts the 7-s measurement if it detects user movement. After 20 levels, the data collection will automatically terminate. This incremental increase serves to subsample the blood pressure oscillogram using the 20 discrete applied forces as points of clean data (Supplement Video 2).

### Cost

A key aspect of our approach is the ultra-low cost nature of the design. Because the mechanism solely relies on a spring for force induction and uses a lens-less optical design to pair with the smartphone, the clip only requires massively manufacturable mechanical parts. Currently, in small-batch manufacturing of 1000 units, the material cost of each of our proposed BPClip system is under $1 USD. The cost consists of the spring ($0.03), the acrylic cover ($0.001), two O-rings ($0.04), the anti-slip pads ($0.03), the metal rod and tube ($0.15) and the resin 3D printed material ($0.55), totaling $0.80. Even at low production, our proposed system is substantially less expensive than any commercial blood pressure monitor. With the majority of the cost from 3D printed plastic, this cost will be substantially lowered at scale with injection molding and bulk component costs.

### Accuracy

**Figure 4:**
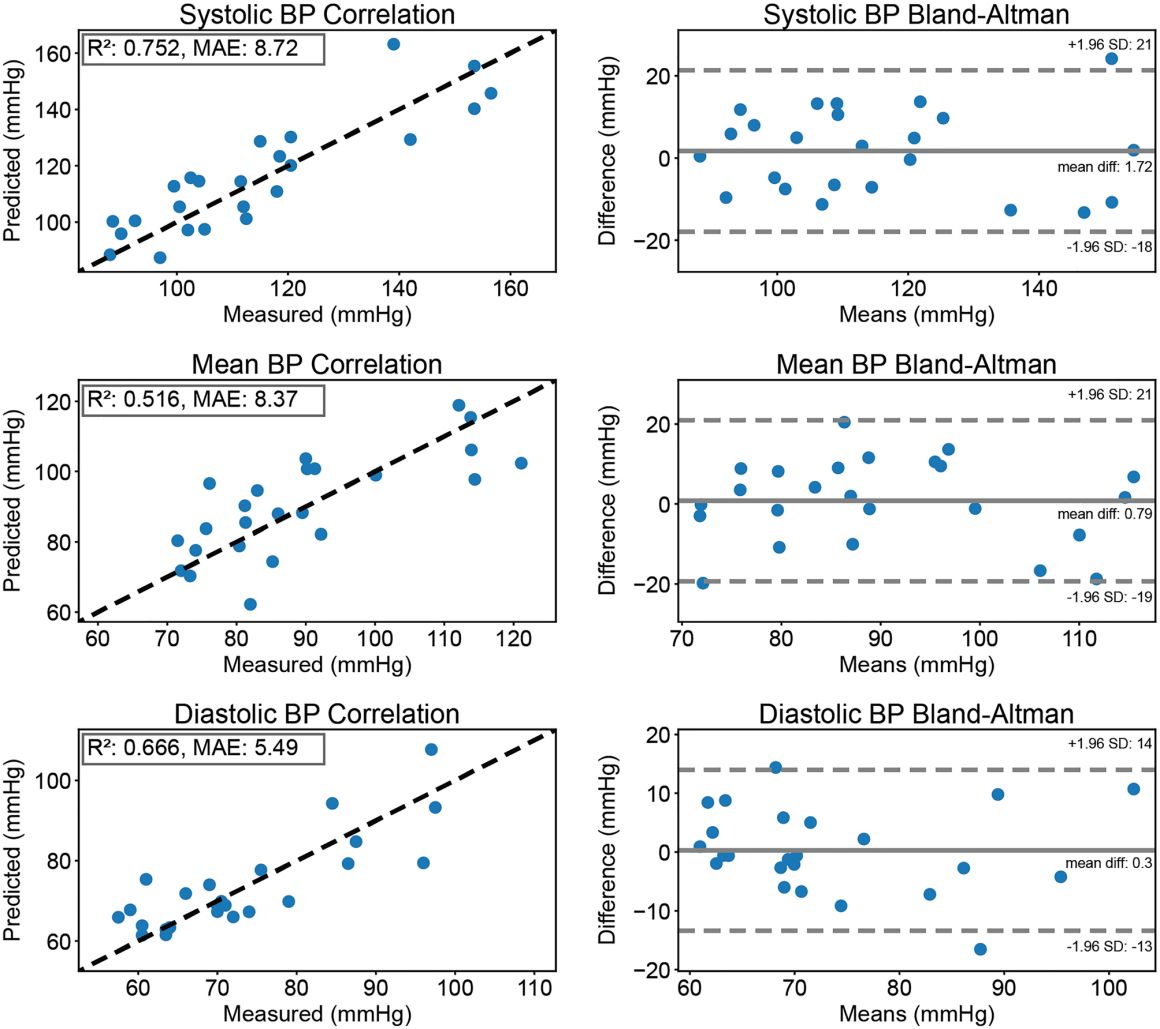
Blood pressure estimation accuracy: accuracy of blood pressure estimation with leave-two-subjects-out validation (12-fold cross-validation with N = 24 subjects) shown in correlation and Bland–Altman plots. The dashed lines in correlation plots represent the best-fit line, where the measured value equals the predicted value.

The accuracy of BPClip is validated on data collected from 24 users with SBP ranging from 80-156 mmHg and DBP ranging from 57–97 mmHg. Figure 4 shows the correlation and Bland–Altman plots for the systolic, mean, and diastolic BP measurements. The systolic and diastolic reference measurements were recorded using a standard arm cuff device. The mean BP reference is calculated from the reference measurements as $meanBP\, (MBP)\ =\ 1/3 \ SBP + 2/3 \ DBP$^32^. BPClip’s measurement achieved a mean absolute error (MAE) of 8.7 ± 10.0, 8.4 ± 10.3, and 5.5 ± 7.0 mmHg and bias of 1.72, 0.79, and 0.3 mmHg for systolic, mean, and diastolic BP, respectively. Five subjects were excluded from analysis for the estimation accuracy analysis due to either poor perfusion which resulted in the inability of the system to acquire pulse data (N = 4), or an atypically large difference between SBP and DBP of greater than 80 mmHg (N = 1). In order to investigate if the result is biased based on skin tone, we further performed a subgroup analysis on the accuracy within different ethnic groups. For SBP, the MAE for Asian, Hispanic, and White groups are 7.7 ± 4.2, 8.6 ± 5.2, and 10.2 ± 6.9 respectively. For DBP, the MAE for Asian, Hispanic, and White groups are 4.5 ± 4.6, 9.5 ± 6.4, and 6.5 ± 2.9 respectively. After running a one-way ANOVA test on these three groups, we found the p-values to be 0.61 and 0.62 for SBP and DBP respectively, indicating there is no significant difference in MAE among the three ethnic groups.

### Usability

Because using BPClip requires the user’s active interaction, we analyzed how much time it takes for a user to complete one measurement. The time spent in each trial was calculated from metadata for each participant. The minimum possible time for a user to complete a measurement is approximately 140 s for the measurement, given 7 s per level for 20 levels if the user completes each level without repeating.

On average, it took 251 ± 127 s for the first trial and 212 ± 45 s for the second trial. Because the right index finger is used for BPClip, we also recorded if the participant was left-handed or not. Among the recruited participants, 6 were left-handed, but none reported difficulty using BPClip. A t-test suggested that the difference in time to complete one measurement trial is not significant between left-handed users (N = 8) and right-handed users (N = 21) (p-value = 0.85).

## Methods

### Study design

29 study participants were recruited from UC San Diego main campus and UC San Diego Medical Center (Details in Appendix A, B) (SBP: 116.8 ± 20.3 mmHg, $\le$ 110 mmHg: N = 12, 110–130 mmHg: N = 10, $\ge$ 130 mmHg: N = 7; DBP: 73.5 ± 12.3 mmHg, $\le$ 70 mmHg: N = 12, 70–80 mmHg: N = 9, $\ge$ 80 mmHg: N = 8) Informed consent was obtained from all participants. All the methods included in this study are approved by the University of California, San Diego Institutional Review Board (IRB) (IRB Approval #804668), and all methods included in our study are in accordance with the relevant guidelines. After being recruited for the study, participants were asked to warm their hands with heat packs to promote blood flow to the fingertips. The participants then received instructions on how to use the device and performed a practice measurement using BPClip. During the practice measurement and all subsequent measurements, the participant was positioned in a seated position with their hand resting on a table surface at the level of their heart, such that both the left bicep and right index finger are at the level of the heart. The participants then performed 2 consecutive measurements with BPClip. Immediately before and after BPClip measurement, an automated blood pressure cuff device [Omron, model: BP7350] was used to measure BP on the left arm. The average of the two cuff measurements was used as the reference.

### Prototype: hardware

BPClip is designed with SolidWorks CAD software and printed with a resin 3D printer (Anycubic Photon Mono X with Hard Tough Resin—Black). The clip dimensions are $5.1 \times 4.1 \times 2.3$ cm. The clip is clamped onto the smartphone (Google Pixel 4) using a 3D-printed screw. The clip consists of 3 main components: the base clip, the pressing platform, and the compression spring.

The base clip is a 3D-printed clip-shaped piece that is mounted to the smartphone. It also includes a 3D-printed screw and nut which can tighten to help fix the clip in position. To ensure a firm and non-slip interface, a silicone pad is used to increase the friction between the clip and the phone screen. The bottom piece is flush with the camera and flashlight LED.

The pressing platform is also 3D-printed and wraps around the bottom piece. It comes in contact with the user’s finger and is displaced as the user applies force to it. The contact area is a circular protrusion with a diameter of 10 mm. To ensure an even surface for the finger contact area, clear acrylic pieces are placed over the openings of the illumination slot and pinhole. Two flanges are used to constrain the angle of the index finger. Two notches are used to help users align their index finger with the pinhole.

Interfacing the base clip and pressing platform is a compression spring with a k-constant of 0.49 N/mm, placed in between the pressing platform and base clip. It renders the pressing platform the ability to move up and down, depending on the amount of force applied by the finger. The spring is pre-loaded with a force of 0.1 N. 6.7 mm more space is allowed for the spring to compress, rendering the potential range of force applied to the finger from 0.1 to 3.3 N. With an area of a circle of 10 mm diameter, the pressure would range from 9.5 to 315 mmHg. Because the prototype is constructed with 3D printing, surface finishes are not perfectly smooth compared to injection molded plastic parts. Two pairs of guiding rods and tubes, with lubricants applied, are added to the pressing platform and base clip respectively to allow for smooth uniform displacement.

A light guide and an imaging path are designed to maximize the light transfer and minimize light leakage. The light guides direct the flash into the protrusion and into the finger. To maximize light transfer from the flash to the finger, chrome spray paint [Ultimate Mirror Chrome, Spaz Stix] is applied to the inner surface of the light guide. A pinhole projects the light from the finger onto the camera via the imaging path. At the end of the light guide and imaging path, two O-Rings were respectively padded in between the bottom piece and the phone to reduce the external light influence.

### Prototype: software

We developed an Android smartphone application to be used with BPClip. The application has 3 main features. First, it infers the applied finger force from the size of the pinhole reflected on the image captured by the camera. The pinhole is projected to the camera sensor as a circle. For each frame, the area of the circles is calculated from the number of pixels with a value above 20 (the image is encoded with unsigned 8-bit integers). To account for different baseline values within the circle due to different amounts of blood circulation, a histogram equalization operation was applied to the raw image before area calculation. The diameter of the circle is then calculated from the area of the circle. The force is plotted in real-time in the force plot UI. To avoid unnecessary confusion for the user, the force signal was processed with a low pass filter to remove the PPG artifact before being plotted.

Second, the application can extract the PPG signal from the pixel values within the circle. For each frame, the average value of the pixels within the circle that have a value between 20 and 254 is calculated to be the PPG signal. The PPG is plotted in real-time in the PPG plot UI.

Third, the application has a UI that helps the user maintain the applied finger force at a specific level. This UI consists of a brightness adjustment feature and a feedback feature. Because the brightness depends on the amount of total blood circulation as well as the skin tone, the size of the circle projected onto the camera at each force level changes depending on the person. To adjust for this, a simple brightness adjustment is performed before each measurement. To perform an adjustment, at the beginning of a measurement round, the circle size when the user rests the finger on the clip with no force is recorded (Force Scale 0%), as well as the circle size when the clip is completely pressed down. (Force Scale 100%). By capturing the 0% and 100% circle sizes, the ensuing measurement can be performed in proportion. 20 force levels are then equally divided from 5 to 95%. During the trial, a green line representing the current target force level appears in the force plot. An additional red indicator line showing the current force applied helps guide the user to adjust the applied force to meet the target force. While the applied finger force does not yet match the target, the indicator line remains red. As soon as the force is in the range close to the target (± 2%), the indicator turns yellow. The user is then expected to hold their finger in position to maintain a constant applied force. If the force stays within the range, after 2 s the indicator turns green from yellow, where the data is collected for 5 s. If the force goes out of the range (± 2%) anytime when the indicator is yellow or green, the indicator changes back to red, and any data collected will be discarded. The data for the current force level then needs to be recollected.

### Data preprocessing

This section elaborates on the process of preprocessing raw data recorded on the smartphone. As thoroughly explained in the prototype hardware section, the camera images contain a pinhole projection. The diameter of the pinhole projection corresponds to the force exerted on the finger. The pixel intensity fluctuations of the pinhole projection correspond to blood flow through the palmar arch artery.

For the data analysis, we only use data captured during the 5-s intervals at the specified force levels from the smartphone application. For the applied force, this smartphone application ensures ± 2% tolerance on each force level. At each of the 20 force levels, the 5 s of recording allows for approximately 3–7 complete pulses of blood flow, as measured by the pixel intensity of the pinhole projection. For each of these force levels, we applied a 0.5–10 Hz band pass filter and then averaged the peak-to-trough amplitude of all complete pulses using peak prominence. This results in one average peak prominence value for each of the 20 force levels. These 20 points make up the subsampled blood volume oscillogram used to calculate blood pressure.

During the data preprocessing, we excluded participants for the following reasons:

- The participant did not have sufficient perfusion to create a PPG signal with significant enough prominence values for interpretation (N = 4).
- The participant had abnormal blood pressure with more than 80mmHg difference between the reference systolic and diastolic BP measurements (N = 1).

This resulted in N = 24 participants for the data analysis and validation steps of the study.

### Blood pressure estimation

This section describes our process of using preprocessed data to predict blood pressure. The preprocessed data contains a 20 point oscillogram of force versus blood volume measurements, where blood volume is measured by PPG prominence. We exclude the lowest and highest force levels of the oscillogram, because when at the lowest force, the finger can sometimes leave the clip, and at the highest force, the spring is fully compressed and the applied finger force may exceed the spring load. From the remaining 18 oscillogram points, we normalize the points between 1 and 0.

The blood pressure estimation consists of an estimation of mean and systolic BP followed by a subsequent estimation of diastolic BP. For the mean and systolic BP estimation, we use the 18 oscillogram points as the input into a Least Absolute Shrinkage and Selection Operator (LASSO) Regression^33^ to estimate systolic and mean BP. The predicted systolic and mean BP will be the input features for the diastolic BP estimation. For estimating diastolic BP, we use a linear regression model, where the only input features are the predicted systolic and mean BP values. For all BP prediction validation results, we use a leave-two-subjects-out validation (12-fold cross-validation) with N = 24 participants. This leave-two-subjects-out validation involves training the BP prediction models on 22 subjects and then predicting the BP for the 2 unseen participants. This process is repeated 12 times so each participant is in the holdout set exactly once.

## Discussion

A key finding in our work is that there is no need to perform a per-user or per-device calibration. This is enabled by the simple top-bottom brightness adjustment that is included at the beginning of a measurement. By asking the user to hold the clip at the furthest compression and then once again at the closest compression, our system adjusts for the total brightness as reflected by a person’s finger. Thus, no matter the skin tone (where darker would absorb more light) or with a dimmer flash (as not all phones have the same brightness), the brightness adjustment equalizes the proportional amplitude of the PPG accordingly.

The consistency of the spring constant is important, however, and cannot be calibrated for by the end-user. With commercial springs typically with a tolerance of ± 10%^34^, it is possible to introduce highly biased measurements simply due to spring variance. Although it is possible to reduce this tolerance issue through quality assurance testing and selecting springs that meet a more stringent tolerance, we anticipate that this may increase the end cost significantly due to low yield. We instead would envision that a manufacturing pipeline would include calibrating an assembled clip using a mechanical testing system to obtain the force to pinhole projection as a calibrated parameter. A QR-code sticker, much like that of a quality assurance sticker will be placed on the device. For the user, the app would instruct them to scan the QR code and the calibration would automatically be downloaded for the clip. This QR generation, at production, can foreseeably be fully automated.

As with all optical-based pulse sensing systems, skin tone needs to be considered. Darker skin tones can absorb more light, thus leading to less total light reflected. Although we did not perform a formal skin tone study, our approach is not significantly affected by skin tone, except for signal strength, which can be addressed through digital amplification by adjusting the sensor sensitivity. Unlike calculating oxygen saturation (SpO$_2$), our method does not depend on the distribution of absorbed light at different wavelengths, but rather only depends on the total amplitude of the signal. Our current prototype sufficiently addresses this issue by chrome-painting the light guide that channels a significant amount of light from the phone flash. This is made possible because smartphones can increase the sensitivity of the camera without compromising the sampling rate. However, increasing the exposure time is not a viable solution, as it would interfere with the pulse signal’s sampling rate.

Through this feasibility study, we identified several limitations of our system that need to be considered for future development of this concept, moving towards practical application.

Our method requires adequate blood circulation in the finger to capture a strong pulse signal. To ensure an adequate amount of blood circulation in the finger, we provided a hand warmer for participants to warm their hands prior to measurement. Before data collection, we checked the participants’ perfusion index (PI) using a pulse oximeter [NuvoMed, A310]. If the PI was $\le$ 1%, participants were instructed to continue warming their hands until their PI rose above 1%. In our validation, 4 participants, even after hand warming, had insufficient blood flow that resulted in no detectable pulse. As a result, the BPClip system could not determine an oscillogram measurement on these participants. Further studies are needed to investigate additional mechanisms to improve blood perfusion for those with poor circulation for use with our system.

A key contribution of our work is that the design of this ultra-low cost blood pressure monitoring attachment is suitable for almost all smartphones on the market. Although the studies presented in this paper were conducted using the Google Pixel 4 smartphone, BPClip relies only on the smartphone camera and flashlight, making our approach suitable for most phones. BPClip can be adapted to other smartphone models with minimal changes, as most phone models would only require adjustments in the distance between the flashlight and the camera. Our current design already takes that into account and uses a bend in the light guide to bring the flashlight to the finger from various distances. We surveyed the distances of flashlights to the closest camera lens from 50 smartphones out of 10 brands, across various pricing ranges (Appendix C). According to the distribution of the flash to camera distances, we characterized the amount of light energy lost when light travels through the light guide with various distances using an optical power meter [Newport 843-R, 818-ST2/DB] (Appendix D). Our findings revealed that the chrome paint inside the light guide significantly reduced light loss, allowing the light guide with chrome paint to function for most phones with a flashlight-to-camera distance of up to 16 mm. In future refinement of the concept, we believe it would also be possible to design a concept that relies on the front facing camera and screen illumination to achieve the same design that is more universal across all phones. However, due to the lower illumination of the screen compared to the flash LED, future designs in this form would require additional care around how to capture enough light from the screen to fully illuminate the finger enough for all skintones.

Compared to a fully automated cuff, a limitation of our system is that users must have a reasonable level of finger dexterity to use BPClip, as they need to hold their index finger steadily while exerting various amounts of force. Therefore, although we have found that our middle-aged participants had no trouble using the device, for some older adults with weakened grip and/or hand tremors, it can be difficult to use BPClip. A future design could consider using a higher stiffness spring to allow for the same range of force application with less compression, resulting in easier force application overall. With smaller displacements we suspect that it will also be easier to perform a continuous force application with less motion artifact, which will be considerably faster to perform than the current 20 level measurement. Moreover, future designs that leverage the front-facing camera/screen could allow users to operate the device with their thumb, which is typically stronger and more dexterous for most people, making the device easier to use compared to controlling actuation with the index finger. This design would require modification to the pinhole design, such as including a low-cost lens, to focus the image to improve imaging resolution to offset smaller displacements of the spring.

Finally, our system performs blood pressure measurements at the digital artery at the finger. Although we demonstrate in our feasibility study that the measurement made at the finger correlates well with that measured at the brachial artery using an automated cuff, there are a number of factors that may affect this end measurement. We suspect that finger callus and other conditions that affect the finger will lead to changes to the finger skin surface mechanics which will distort the force applied by the device to the finger artery. This will likely lead to inaccuracies. Therefore, it is important for future studies to incorporate varying finger conditions, such as callused fingers, vasoconstricted fingers, deformities, and size variations, in order to fully characterize how these differences affect the measurement. This will help determine whether additional calibrations or models are needed to address such variation and maintain accuracy across people.

## Conclusion

Health monitoring should be available to everyone, no matter their country of origin, their skin tone, their gender, and most of all, their income. In this paper, we propose a solution to democratize blood pressure (BP) monitoring by converting billions of smartphone cameras, even the cheapest ones, into BP monitors with an ultra-low-cost plastic clip that can be produced at-scale for mere cents. Our current prototype’s material costs less than $1 USD to manufacture at low quantities, but at scale, we expect that a blood pressure monitor will reach an even lower cost.

## Supplementary Information

Supplementary Information 1.

Supplementary Information 2.

Supplementary Information 3.

Supplementary Information 4.

Supplementary Video 1.

Supplementary Video 2.

Supplementary Legends.

## Data Availability

The data that support the findings of this study are available from the corresponding author upon reasonable request.

## Code availability

The code that supports the findings of this study is available from the corresponding author upon reasonable request.
