# Supplementary material for: Ultra-low-cost mechanical smartphone attachment for no-calibration blood pressure measurement
Source: Sci Rep. 2023 May 29;13:8105. doi: 10.1038/s41598-023-34431-1 (PMC10227087; doi:10.1038/s41598-023-34431-1)
Supplement: Supplementary file 1 — Supplementary Information 1. [file 41598_2023_34431_MOESM1_ESM.pdf]

Appendix A Participants Demographic Information

	N	Min	Max	Mean	STD
Total Subjects	29				
Male	21				
Female	8				
Age		18	56	30.2	12.3
Height (cm)		152	191	173.9	9.5
Weight (kg)		50	105	69.3	14.5
Race/Ethnicity					
Asian	13				
White	9				
Hispanic	7				
SBP (mmHg)		88	156.5	116.8	20.3
≤ 110	12				
> 110 & < 130	10				
≥ 130	7				
DBP (mmHg)		57.5	97.5	73.5	12.3
≤ 70	12				
> 70 & < 80	9				
≥ 80	8				
