# Supplementary material for: Ultra-low-cost mechanical smartphone attachment for no-calibration blood pressure measurement
Source: Sci Rep. 2023 May 29;13:8105. doi: 10.1038/s41598-023-34431-1 (PMC10227087; doi:10.1038/s41598-023-34431-1)
Supplement: Supplementary file 2 — Supplementary Information 2. [file 41598_2023_34431_MOESM2_ESM.pdf]

Appendix B Individual Participant Information

ID	Sex	Age	Ethnicity	Height (cm)	Weight (kg)	Est.SBP (mmHg)	Est.DBP (mmHg)	Ref.SBP 1 (mmHg)	Ref.DBP 1 (mmHg)	Ref.SBP 2 (mmHg)	Ref.DBP 2 (mmHg)	Handed ness	Tech. Literacy	Trial 1 Time (s)	Trial 2 Time (s)
1	F	54	Hispanic	152	78	*	*	147	65	147	60	right	7	566	230
2	M	56	White	191	98	145.7	93.3	154	98	159	97	right	8	249	201
3	M	18	White	185	70	87.4	66.0	95	68	99	76	right	8	220	217
4	M	23	Asian	185	73	101.2	61.4	112	61	113	60	right	9	193	200
5	F	22	Asian	164	50	95.9	62.9	91	64	89	63	left	9	237	207
6	M	20	Asian	165	51	105.5	69.8	111	82	113	76	right	7	226	228
7	M	28	Asian	175	60	100.3	75.4	88	61	89	61	right	10	201	192
8	F	27	Asian	167	55	97.2	67.3	99	75	105	73	right	8	186	207
9	M	21	Asian	168	52	105.4	67.8	100	61	101	57	right	9	193	187
10	M	25	White	170	59	100.5	65.9	95	60	90	55	right	10	191	182
11	M	18	Hispanic	163	59	110.9	69.9	122	72	114	69	right	7	199	203
12	M	20	White	188	80	**	**	100	68	108	64	right	9	196	191
13	M	21	Asian	180	68	114.5	71.8	109	64	107	60	left	8	205	192
14	F	20	Asian	172	54	88.4	61.5	90	62	86	65	right	9	216	203
15	M	22	Asian	183	75	123.4	68.9	115	68	122	74	right	8	204	188
16	M	24	Asian	168	54	112.7	63.8	98	60	101	61	left	9	281	249
17	M	26	White	180	85	128.7	77.7	117	78	113	73	right	8	178	191
18	M	24	White	183	78	114.4	74.0	110	73	113	65	right	8	188	188
19	M	21	Asian	188	65	97.5	63.4	106	65	104	63	right	8	229	197
20	F	26	Asian	170	60	**	**	103	72	102	68	right	8	182	191
21	M	27	Asian	179	65	115.8	68.8	114	76	119	77	right	9	185	167
22	F	28	Hispanic	165	61	130.2	79.3	122	88	119	85	left	10	191	190
23	F	28	Hispanic	162	60	**	**	145	98	139	94	left	10	239	177
24	M	30	Hispanic	173	68	**	**	124	73	117	67	right	8	253	200
25	M	44	Hispanic	170	80	129.3	79.5	134	77	125	77	right	8	210	222
26	M	47	White	178	79	120.1	67.3	140	94	146	101	right	10	244	212
27	M	55	Hispanic	172	68	140.2	84.8	152	86	155	89	right	6	714	411
28	F	51	White	163	105	155.4	94.3	142	84	165	85	left	8	277	213
29	M	51	White	185	100	163.2	107.7	130	92	148	102	right	7	206	200

SBPsgreaterthan80mmHgfromDBP

***PPG too low due to poor perfusion*
