# Supplementary material for: Ultra-low-cost mechanical smartphone attachment for no-calibration blood pressure measurement
Source: Sci Rep. 2023 May 29;13:8105. doi: 10.1038/s41598-023-34431-1 (PMC10227087; doi:10.1038/s41598-023-34431-1)
Supplement: Supplementary file 3 — Supplementary Information 3. [file 41598_2023_34431_MOESM3_ESM.pdf]

Appendix C Survey of the Distance of Smartphone Flashlight to the Closest Camera

Brand	Model	Flash to Camera Distance
Samsung	Galaxy A53 5G	17.8
Samsung	Galaxy M53	13.3
Samsung	Galaxy S21 FE 5G	14.9
Samsung	Galaxy Z Flip3 5G	12.3
Samsung	Galaxy Z Fold3 5G	10.0
Motorola	Moto G53	11.2
Motorola	Edge 30	10.1
Motorola	Moto X40	12.5
Motorola	Edge 30 Ultra	14.9
Motorola	ThinkPhone	11.6
LG	W41 Pro	10.6
LG	Velvet	9.2
LG	Stylo 6	10.2
LG	Q92 5G	10.0
LG	K62	10.5
Google	Pixel 4a	12.4
Google	Pixel 5a 5G	9.7
Google	Pixel 5	9.3
Google	Pixel 7	16.5
Google	Pixel 4	10.0
Xiaomi	Poco X5 Pro	16.3
Xiaomi	Redmi Note 12 Pro	12.2
Xiaomi	12T Pro	12.3
Xiaomi	Mi 10 Ultra	8.5
Xiaomi	Mi 11	12.9
OnePlus	Nord CE 2 5G	7.2
OnePlus	Nord CE 2 Lite 5G	9.8
OnePlus	Nord 2 5G	8.0
OnePlus	8 5G UW	10.1
OnePlus	8T	9.9
Nokia	G60	10.8
Nokia	2.4	8.5
Nokia	X30	9.9
Nokia	XR20	11.4
Nokia	3 V	9.6
HTC	Desire 20+	10.0
HTC	Desire 21 Pro 5G	8.9
HTC	Desire 22 Pro	11.7
HTC	Desire 20 Pro	10.7
HTC	Wildfire E plus	12.2
VIVO	iQOO Z7	19.0
VIVO	V21	10.5
VIVO	iQOO 9 SE	11.1
VIVO	X70	12.6
VIVO	X50 Pro+	10.2
Apple	iPhone 14 Pro Max	13.3
Apple	iPhone 13	14.6
Apple	iPhone 12	14.0
Apple	iPhone SE (2022)	12.6
Apple	iPhone 13 Pro	13.0

Source: https://www.gsmarena.com
