# Supplementary material for: Ultra-low-cost mechanical smartphone attachment for no-calibration blood pressure measurement
Source: Sci Rep. 2023 May 29;13:8105. doi: 10.1038/s41598-023-34431-1 (PMC10227087; doi:10.1038/s41598-023-34431-1)
Supplement: Supplementary file 4 — Supplementary Information 4. [file 41598_2023_34431_MOESM4_ESM.pdf]

Appendix D Light Guide Performance

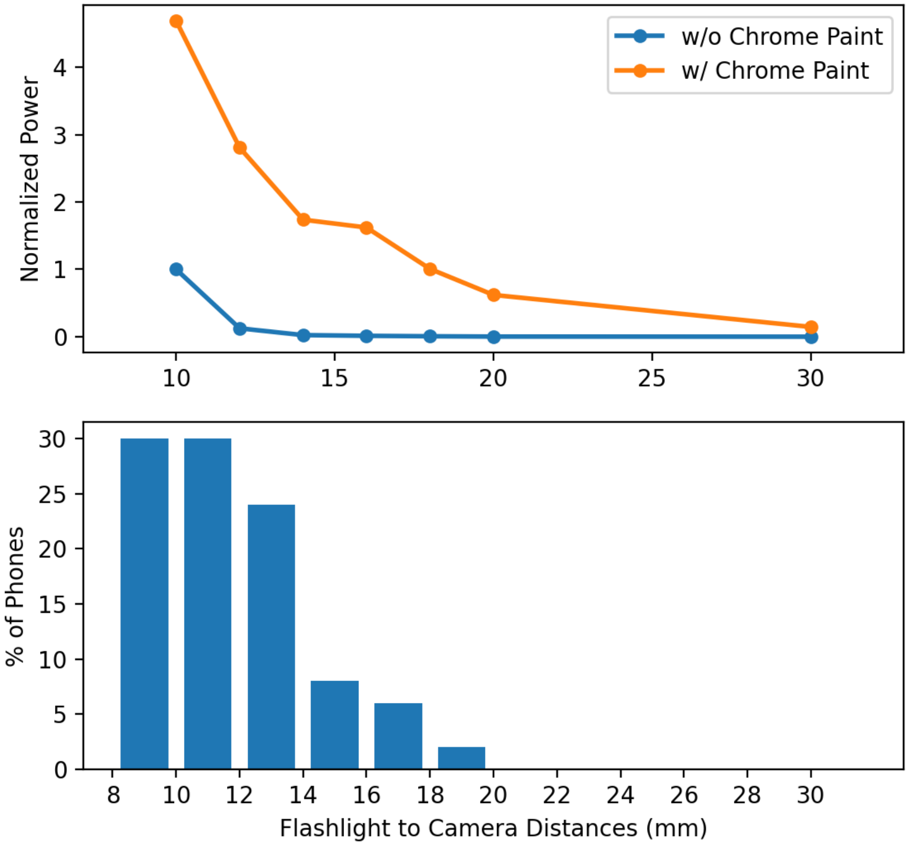

Fig. D1 **Top**: Normalized light power loss as light travels through the light guide with various lengths, mimicing the light guide's performance with smartphones of various flash to camera distances. **Bottom**: The distrubution of the distance of a flashlight to its closest camera.
