# Supplementary material for: Ultra-low-cost mechanical smartphone attachment for no-calibration blood pressure measurement
Source: Sci Rep. 2023 May 29;13:8105. doi: 10.1038/s41598-023-34431-1 (PMC10227087; doi:10.1038/s41598-023-34431-1)
Supplement: Supplementary file 7 — Supplementary Legends. [file 41598_2023_34431_MOESM7_ESM.docx]

Supplement Video

1. File name: concept_main_6.mp4
   1. Title: Introduction to the working principle of the BPClip
   2. Summary: A video representation of Section 2.1
2. File name: demo_main_5.mp4
   1. Title: Demonstration of how to use BPClip
   2. Summary: A video representation of Section 2.3

Supplement Table

1. Participant Demographic Information
2. Individual Participant Information
